# Results of the META-Health Study Suggest Pathways by Which Vitamin D Affect Obesity and Cardiovascular Risk through Adiponectin Levels may Require Further Characterization in Subgroups

**DOI:** 10.3389/fpubh.2014.00269

**Published:** 2014-12-08

**Authors:** Tony Kuo

**Affiliations:** ^1^Division of Chronic Disease and Injury Prevention, Los Angeles County Department of Public Health, Los Angeles, CA, USA

**Keywords:** vitamin D, adiponectin, obesity, cardiovascular risk, publication bias

Recent review of Bidulescu and colleagues’ article on the association between vitamin D and adiponectin and its relationship with body mass index in African-Americans, as compared to Whites, reinforced the value of conducting stratified analyses using more resolute subpopulation/subgroup data (1). Although potential mechanisms were carefully considered and described by the investigators of the META-Health Study, the mixed results raised questions about how vitamin D and adiponectin interact in the presence of normal weight versus obesity. For example, the authors hypothesized that the inverse association between vitamin D and adiponectin among lean African-American women may be due to a possible reduced negative regulation of the adipose-tissue renin-angiotensin system by vitamin D metabolites. While this explanation has some support in the literature (2, 3), the significant direct association between vitamin D and adiponectin among lean White women and the lack of association found among obese individuals suggest biological pathways by which vitamin D affects adiponectin expression may be more complex in subgroups and may require further characterization. Prior studies have indicated that adiponectin secretion is paradoxically decreased in obesity, likely as a result of the inhibitory effects of inflammatory factors secreted by hypertrophic adipocytes (i.e., as in obesity) (3–5). Taken together, the results of the META-Health Study suggest that further research may be needed to characterize how vitamin D actually mediate obesity and cardiovascular risk through adiponectin levels by race, gender, and body mass index categories. As indicated by the authors, these study data, in spite of their inherent limitations, could be used to inform if and how randomized controlled trials and other studies on vitamin D supplementation should be conducted. An added value of Bidulescu et al.’s investigation is the contribution of mixed results to the evidence base in this field. All too often, publication bias impedes release of these kinds of null or mixed result findings, especially as they relate to subpopulations or subgroups with high burden of obesity, diabetes, and/or cardiovascular disease. In the “real” world setting, e.g., in health policy and clinical practice, negative or mixed results frequently have as much of an impact as positive results on decision-making.

## Conflict of Interest Statement

The author declares that the research was conducted in the absence of any commercial or financial relationships that could be construed as a potential conflict of interest.
